# Supplementary figures and images for: Combinatorial treatment increases IKAP levels in human cells generated from Familial Dysautonomia patients
Source: PLoS One. 2019 Mar 19;14(3):e0211602. doi: 10.1371/journal.pone.0211602 (PMC6424424; doi:10.1371/journal.pone.0211602)

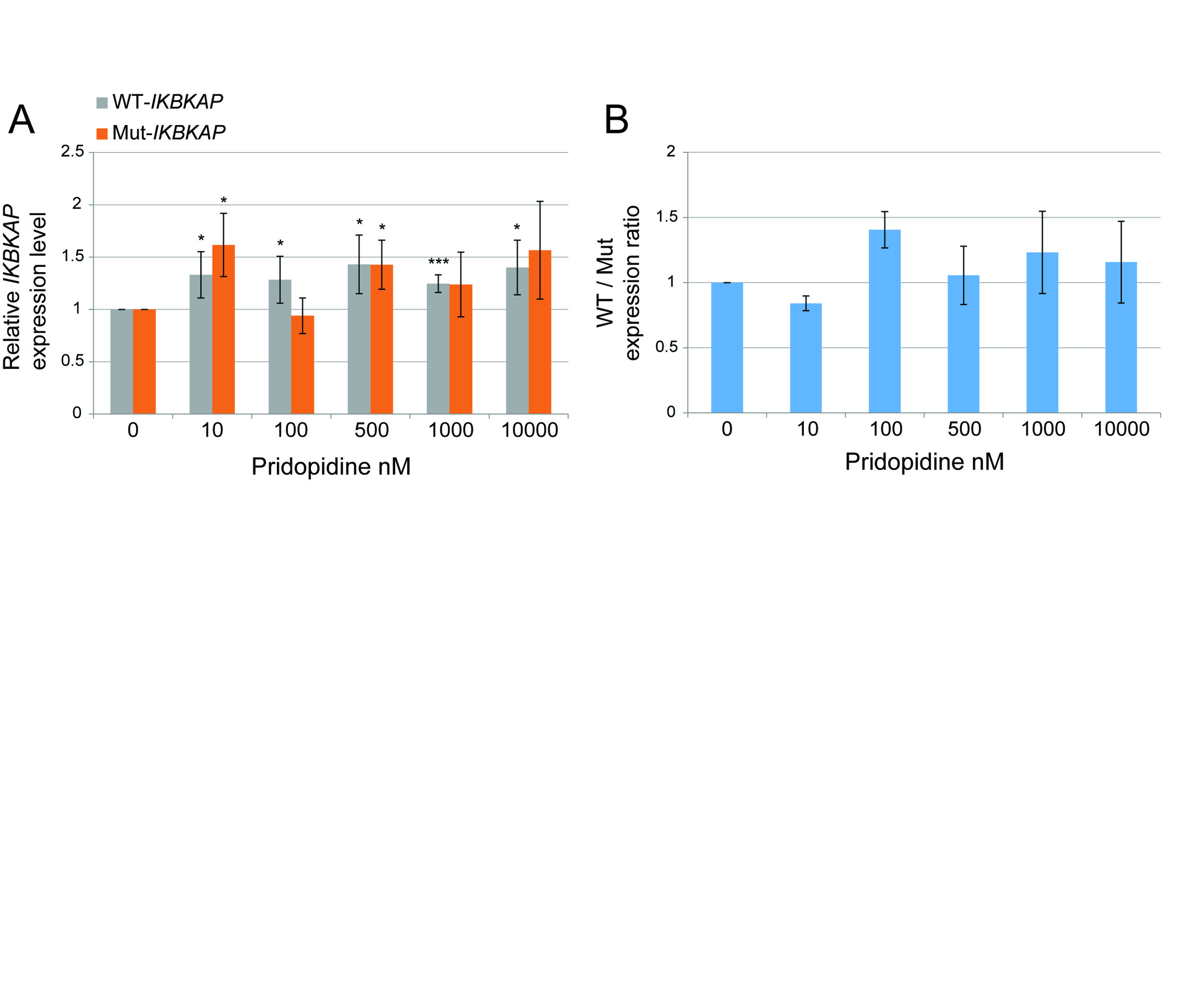

Supplement: S1 Fig — FD cells were treated with 0, 10, 100, 500, 1000 and 10,000 nM of Pridopidine, 5 days later RNA was extracted. (A) Relative IKBKAP expression level of the WT and Mutant isoforms, analyzed by qRT-PCR. (B) Ratio of WT and Mutant IKBKAP isoforms relative expression, analyzed by qRT-PCR. Asterisks denote statistically significant differences (*P ≤ 0.05, and ***P ≤ 0.005) relative to control (vehicle only); Student’s t-test. (TIF) [file pone.0211602.s001.tif]

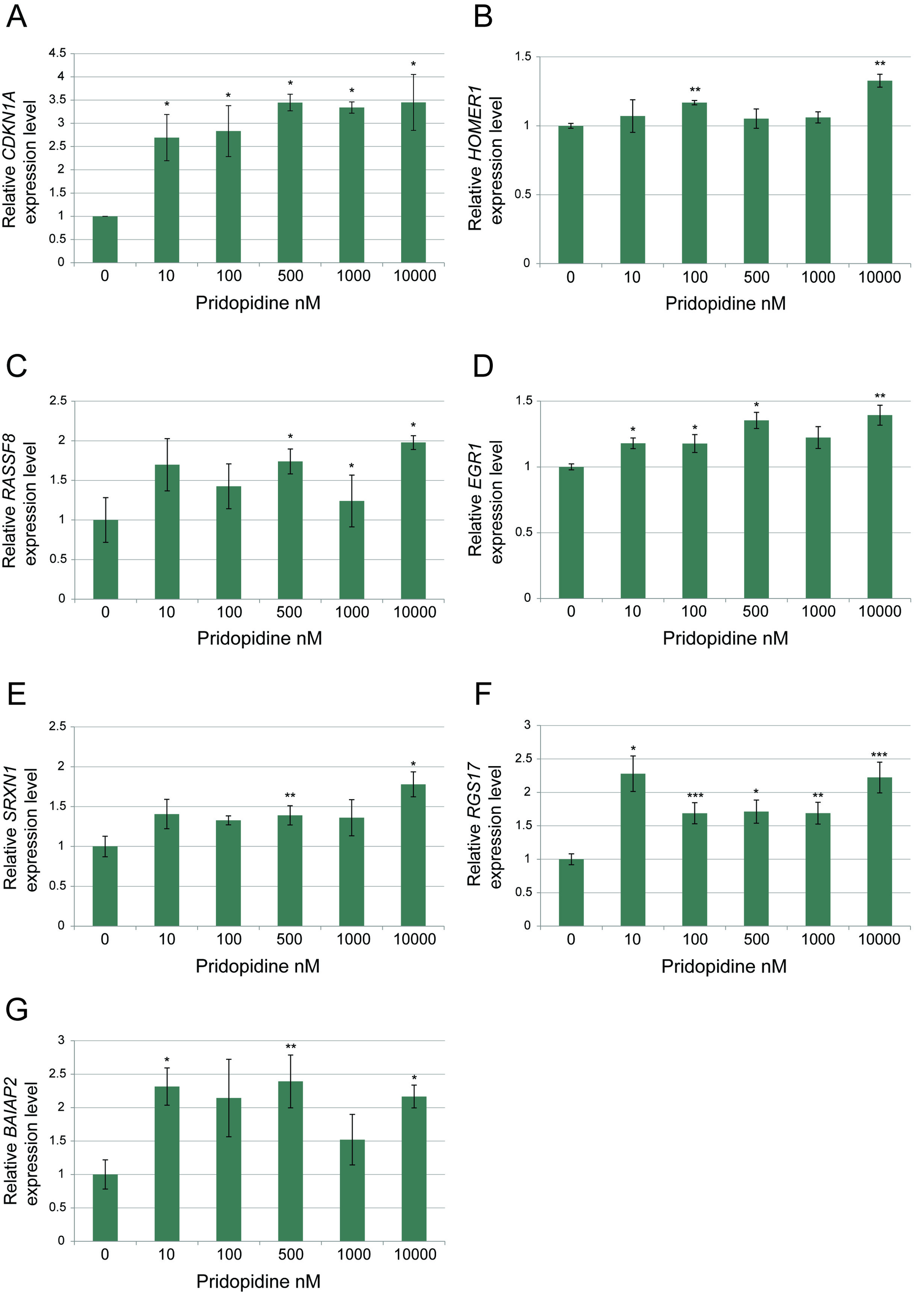

Supplement: S2 Fig — FD cells were treated with 0, 10, 100, 500, 1000 and 10,000 nM of Pridopidine. RNA was extracted 5 days after treatment and qPCR was used to quantify (A) CDKN1A, (B) HOMER1, (C) RASSF8, (D) EGR1, (E) SRXN1, (F) RGS17, and (G) BAIAP2 mRNA transcripts. All values were normalized to LZIC. Asterisks denote statistically significant differences (*P ≤ 0.05, **P ≤ 0.01, and ***P ≤ 0.005) relative to control; Student’s t-test. (TIF) [file pone.0211602.s002.tif]
