# Supplementary material for: Combinatorial treatment increases IKAP levels in human cells generated from Familial Dysautonomia patients
Source: PLoS One. 2019 Mar 19;14(3):e0211602. doi: 10.1371/journal.pone.0211602 (PMC6424424; doi:10.1371/journal.pone.0211602)
Supplement: S1 Table — (DOCX) [file pone.0211602.s003.docx]

**S1 Table. List of primers.**

| **Gene** | **Forward primer** | **Revers primer** |
| --- | --- | --- |
| WT-*IKBKAP* | TTCACGGATTGTCACTGTTGTG | TGTCCAACCACTTCCGAATCTG |
| Mut-*IKBKAP* | CAAAGCTTGTATTACAGACTTATGTTTAAAG | CTTAGGGTTATGATCATAAATCAGATTG |
| *LZIC* | TGATACAGATGAATATGAAGAAACC | TCTACCAAAGTCATATTTCCAGAC |
| *CDKN1A* | CTGTCTTGTACCCTTGTGCC | GGTAGAAATCTGTCATGCTGG |
| *HOMER1* | GGCTCAAAGGCAATAATAAATAGTA | GAAAGATGATGCTCAGAGGAG |
| *RASSF8* | GATCGGTAAGGTCAAAGGGG | GTTCTTTGTCCTGTAAGCGTTTG |
| *EGR1* | GACCGCAGAGTCTTTTCCTG | CTGTTGGGTGCAGGCTCC |
| *SRXN1* | CAC GAT CCG GGA GGA CC | TCT CGC TGC AGT TGC TGG |
| *RGS17* | GGT CCT GAA GTA GCT GAA ATG | CAC AGT GAG GCA GGA GCA |
| *BAIAP2* | CTG GCA GGT GTG ACG TAT G | GAT CTG CCT GTG GAC TTC AG |
